# A new paradigm for drug discovery in the treatment of complex diseases: drug discovery and optimization

**DOI:** 10.1186/s13020-025-01075-4

**Published:** 2025-03-24

**Authors:** Yu Yuan, Lulu Yu, Chenghao Bi, Liping Huang, Buda Su, Jiaxuan Nie, Zhiying Dou, Shenshen Yang, Yubo Li

**Affiliations:** 1 State Key Laboratory of Component-Based Chinese Medicine, Tianjin University of Traditional Chinese Medicine, Tianjin, 301617 China; 2 Collaborative Innovation Center of Mongolian Medicine, Inner Mongolia Medical University, Hohhot, 010110 China; 3 School of Traditional Chinese Medicine, Tianjin University of Chinese Medicine, Tianjin, 301617 China

**Keywords:** Natural products, Drug repurposing, Drug discovery

## Abstract

In the past, the drug research and development has predominantly followed a "single target, single disease" model. However, clinical data show that single-target drugs are difficult to interfere with the complete disease network, are prone to develop drug resistance and low safety in clinical use. The proposal of multi-target drug therapy (also known as "cocktail therapy") provides a new approach for drug discovery, which can affect the disease and reduce adverse reactions by regulating multiple targets. Natural products are an important source for multi-target innovative drug development, and more than half of approved small molecule drugs are related to natural products. However, there are many challenges in the development process of natural products, such as active drug screening, target identification and preclinical dosage optimization. Therefore, how to develop multi-target drugs with good drug resistance from natural products has always been a challenge. This article summarizes the applications and shortcomings of related technologies such as natural product bioactivity screening, clarify the mode of action of the drug (direct/indirect target), and preclinical dose optimization. Moreover, in response to the challenges faced by natural products in the development process and the trend of interdisciplinary and multi-technology integration, and a multi-target drug development strategy of "active substances — drug action mode — drug optimization" is proposed to solve the key challenges in the development of natural products from multiple dimensions and levels.

## Introduction

From 2014 to 2023, the US Food and Drug Administration (FDA) approved 456 new drugs [1]. Among them, problems such as high research and development costs, long research cycles, and clinical safety and efficacy have become obstacles to drug research and development. The traditional drug research and development mode usually follows the method of "one drug, one target", which plays a unique therapeutic advantage in the clinic and brings huge clinical benefits [2–4]. It is undeniable that there are some problems in the treatment of single-target drugs, such as insufficient therapeutic effect, adverse side effects (off-target effects, etc.) and increased incidence of drug resistance [5, 6]. The main reason is that when a single target drug interferes with the target or inhibits the downstream pathway, the body produces self-resistance, activates the bypass biological pathway, the mutation of the therapeutic target and the activation/interaction of its upstream and downstream effectors [7–9]. In addition, off-target effects also limit the therapeutic effect of single-target drugs, which can also bring corresponding toxicity when bringing the expected efficacy [10, 11]. A variety of complex molecular networks participate in the mechanism of many common diseases, especially when dealing with complex diseases, single target drugs are difficult to achieve the expected efficacy [12].

The complexity of disease is mainly caused by the joint action of genetic, physiological, environmental and behavioral factors, involving a variety of potential targets and pathways, and including a variety of pathophysiological states in this complex network [13]. Cardiovascular disease, cancer, alzheimer's disease, diabetes, kidney disease and infectious diseases (lower respiratory tract infection, malaria, tuberculosis, HIV/AIDS) and other complex diseases are the main causes of death in the world [14]. These diseases involve highly complex etiologies, leading to intricate pathogenesis, and drugs targeting single targets or single pathogenic pathways are not easy to achieve good therapeutic effects. In addition, the occurrence and development of disease is a dynamic process of development and transformation, and the complexity is also reflected in the complexity of clinical symptoms and prevention means [15]. For example, the sudden disease COVID-19 affects not only the respiratory tract but also the gastrointestinal tract, nervous or cardiovascular system (cough, fever, diarrhea, etc.) [16]. As a result, traditional single target drugs make it difficult to treat multiple symptoms at the same time [17]. In addition, the prevalence of complications during disease progression is on the rise, that is, the presence of one disease usually leads to the occurrence of one or more other diseases, which also increases the complexity and difficulty of diagnosis and treatment [18–20]. This shows that "single-target, high affinity and high selectivity" may not be enough for effective treatment in clinical practice, especially in the face of complex multifactorial diseases, single-target drugs often have poor efficacy or high toxicity.

The proposal of "designed multiple ligands" in 2004 provided a new idea for drug design and discovery, that is, multi-target drugs simultaneously regulate multiple targets and multiple links in the disease network system, affect the overall balance of the body, and then improve the efficacy and safety (reduce toxicity and drug resistance) [21]. Compared with single-target drugs, multi-target drugs have the characteristics of "multi-target, low affinity and low selectivity", which overcome the limitations of single-target drugs from the perspective of total effect and provide positive clinical effects [22]. At present, there are three different treatment methods in the field of multi-target therapy, which are multi-drug combination therapy, fixed-dose drug combination therapy and multi-target drug therapy. The first two multi-target therapies are drug combinations, which can improve the efficacy or reduce the individual dose, reduce side effects and drug resistance through the interaction between multiple drugs (synergy, addition, antagonism), so as to improve the clinical effect [23]. According to historical data, since the first combination drug was approved by the FDA in 1943, drug combinations have become more and more common. In the past decade, FDA has approved more than 80 kinds of combination drugs [24]. It is worth noting that natural products are natural multi-target drugs, which have the characteristics of structural diversity, high multi-target activity and low toxicity, and become an important source of one drug multi-target drugs [25]. Medicinal plants provide a large number of natural products, and a variety of drugs have been found and applied in clinics, such as morphine, paclitaxel, resveratrol, etc. Therefore, the development of drugs from natural products derived from medicinal plants has become a hot research direction of multi-target drugs, and the combination of drugs simultaneously includes a variety of therapeutic methods in the field of multi-target therapy (a variety of multi-target drug combinations).

However, it has always been a difficult problem to develop multi-target drugs with good drug resistance from natural products. The key problems existing in the current natural product research and development strategy are: (1) the material basis of efficacy is unknown; (2) the activity research is not deep enough, and the mechanism of action is not clear; (3) ignoring the formulation of the clinical optimal dose [26–28]. The great progress of modern disciplines has spawned a variety of biological and chemical methods to solve the above problems. Potential natural products were initially screened from natural products based on their own activities or toxic side effects, therapeutic factors, and therapeutic targets [29]. Key technologies such as network pharmacology, integrative omics, CRISPR gene editing, and direct targets were used to clarify the target proteins and mechanism of action of natural products [30]. In addition, dose prediction is performed at many stages of drug discovery and development to achieve drug design [31]. Therefore, the interdisciplinary and technological integration provide new technologies and strategies for the research and development of natural products. However, how to highly integrate interdisciplinary methods has been an obstacle in natural product drug discovery. The purpose of this review is to summarize the strategies and technologies established in the development of natural products from medicinal plants, and propose a multidisciplinary natural product research and development system to solve the key problems in the development of natural products, so as to achieve the goal of "identify components, recognize mechanism and explicit doses".

## Natural product discovery technology

Traditionally, drug development takes over a decade and billions of dollars, with less than 1% of compounds entering clinical trials. However, the safety, dosage, and toxicity characteristics of drugs in drug repurposing strategies are known, greatly improving the efficiency of the drug discovery process [32]. The most representative drugs are aspirin and sildenafil [33, 34]. Therefore, drug repurposing provides new ideas and methods for the treatment of complex diseases, such as artemisinin (*Artemisia annua*) [35], As2O3 (*Arsenic*) [36], bicyclol (*Schisandra chinensis (Turcz.) Baill.*) [37], ephedrine (*Ephedrae herba*) [38], etc. The screening of traditional drug repurposing is often based on clinical experience and observation. With the development of research technology, methods such as phenotype screening (high-throughput screening (HTS) and high-content screening (HCS)), disease target and chemical structure screening (computer-aided drug design (CADD)), and machine learning (ML) have been proposed and applied, effectively improving the possibility of drug repurposing.

### High-throughput screening and high-content screening

**Fig. 1:**
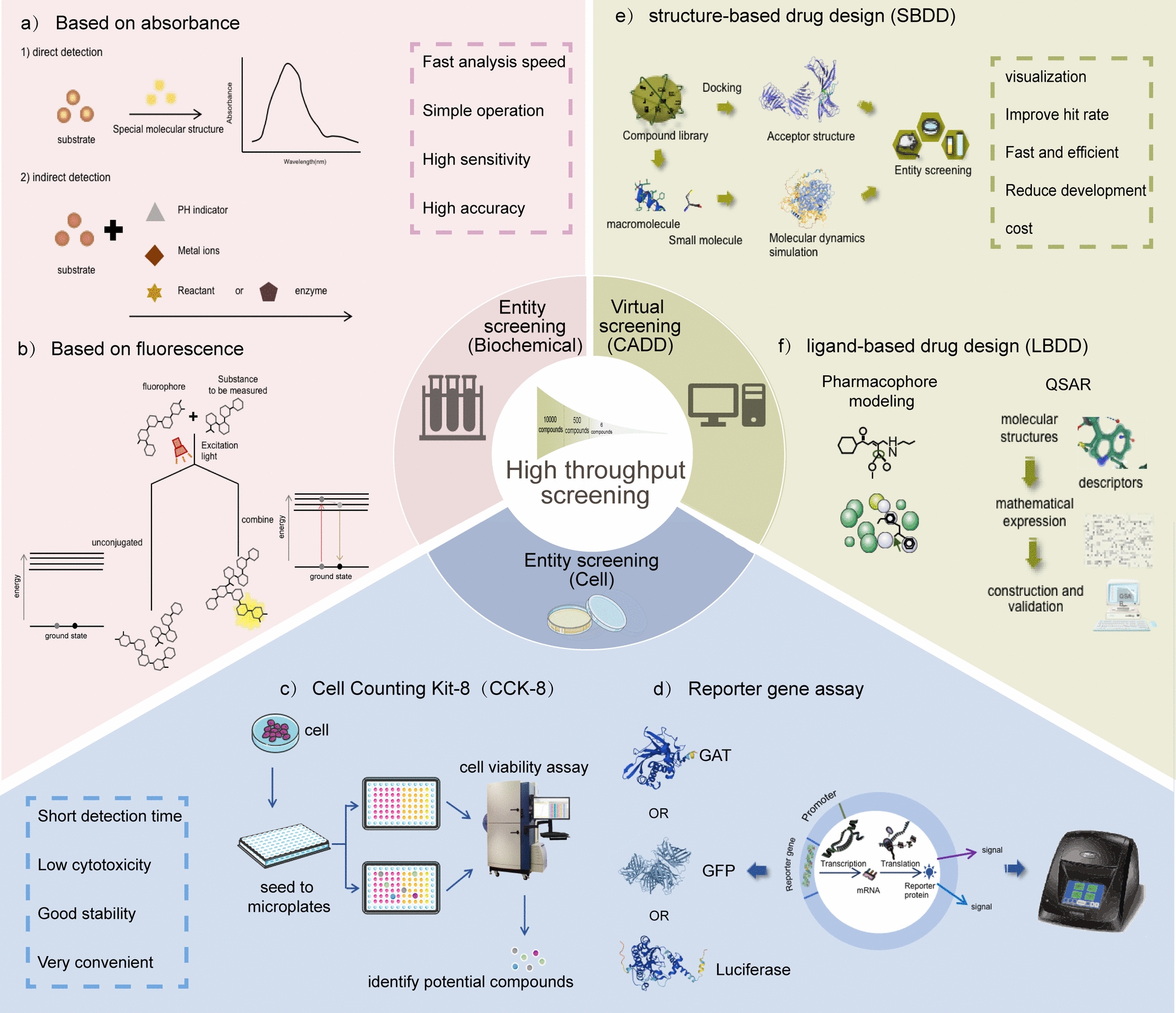
High throughput screening strategy for drugs. **a** and **b** belongs to the biochemical level entity screening methods. **c** and **d** belongs to the cell level entity screening methods. **e** and **f** belongs to the virtual screening methods. PH: Pondus Hydrogenii; CAT: Chloramphenicol Acetyltransferase; GFP: Green fluorescent protein; QSAR: Quantitative Structure–Activity Relationships

The use of de-risk compounds in treating diseases through "drug repurposing" can rapidly develop drugs that can be applied to clinical treatment [39]. HTS technology, based on molecular or cellular level experimental methods, has gradually become a powerful tool for accelerating drug combination therapy research due to its characteristics of trace, fast, sensitive, and efficient. Currently, the commonly used HTS systems are divided into biochemical and cell screening systems [40]. Among them, the biochemical screening system is mainly based on fluorescence or absorbance to detect the binding of purified target proteins to drugs or the impact of enzyme activity (Fig. 1a, b); Cell screening systems typically detect the ability of drug-induced cell phenotypes without knowing the target (Fig. 1c, d). Currently, a large number of studies have utilized this method to the screening of natural products and/or multi-target drugs (Table 1).

**Table 1:** Application examples of high throughput screening techniques

Method	Drug library	Total drugs	Screening quantity	Screening drugs	Disease	Refs.
HTS	Compound library	430,376	378	RU-0415529	SARS-COV-2	[157]
	Small Molecule Libraries	3100	74	(S)-amlodipine besylate	Craniopharyngioma	[158]
	Marine compound library	200	1	Naamidine J	Hepatitis B	[159]
	The HTS customized library	2097	16	Panobinostat	Pituitary neuroendocrine tumors	[160]
	lead-like compound library	1280	8	LMK 235, PI 828, Pyroxamide et al	Medulloblastoma	[161]
	Compound library	3.8 million	392	BAY-593	Breast cancer	[162]
	FDA library	2625	1	Baicalin	Acute ischemic stroke	[163]
	Compound library	137,585	19	Morpholine series and Sulfonylamine series	Tuberculosis	[164]
	purchased from Selleck	227	5	Cepharanthine	Japanese encephalitis	[165]
	FDA-approved drugs	1622	1	Albendazole	Tumor	[166]
	herb extracts	578	20	Ginsenoside F2	Hepatocellular carcinoma	[167]
	Spectrum Collection library	2400	9	Phenylmercuric acetate、Captan、Chloranil et al	Schizophrenia	[168]
	Non-cancer drug	4518	50	Disulfiran、Tepoxalin et al	Cancer	[169]

HCS technology was gradually developed after the rise of HTS technology, also known as cellomics, which is a cell phenotype screening technology combining automatic fluorescence microscopy with automatic image analysis [41]. The experimental process includes 5 parts: cell culture, sample preparation and exposure, image acquisition, image analysis and data mining. On the premise of maintaining the integrity of cell structure and function, HCS realizes the simultaneous detection of multiple targets and parameters (cell morphology and intracellular parameters) of natural products by analyzing the cell image in the porous plate [42, 43]. Compared with HTS, the results obtained by HCS are diversified, and have been applied to the identification of therapeutic compounds for various human diseases, including: cell signaling pathways, oncology, neurobiology, in vitro toxicology, target verification and other fields of life sciences [44–46]. Therefore, HCS can better cope with the complexity of drug research, and more and more studies apply HCS technology to the screening of drug screening (Table 2).

**Table 2:** Application examples of high content screening techniques

Method	Target	Drug library/Drug	Total drugs	Screening quantity	Screening drugs	Disease	Refs.
HCS	Nitroreductase and the red fluorescent protein mCherry	Drug/compound library	138	7	Belinostat	Focal segmental glomerular sclerosis	[170]
	Epithelial mesenchymal transition	MedChemExpress (MCE) compound library	306	5	Camptothecin, dimethyl curcumin, artesunate et al	Fibrotic diseases and cancer	[171]
	Transcriptional repressor Snail	Crude extract from the leaves of X. vielana	–	24	Compound 12	Osteosarcoma	[172]
	Schistosoma mansoni thioredoxin glutathione reductase (SmTGR)	Compound library	29	2	2-[2-(3-methyl-4-nitro-5-isoxazolyl)vinyl]pyridine and 2-(benzylsulfonyl)−1,3-benzothiazole	Schistosomiasis	[173]
	α-SMA, E-cadherin	Chinese herbal medicine Tong-Mai-Yang-Xin-Wan (TMYX)	22	5	Glycyrrhizic acid, glyasperin A, and licorisoflavan A	Chronic kidney disease	[174]
	DNA, F-actin, and microtubules	FDA-approved drugs	55	5	Combination vinblastine/ispinesib	Triple-negative breast cancer	[175]
	B7-H3	FDA-approved drugs	1114	55	Ingenol-3-angelate	Osteosarcoma	[176]
	123 protein targets	Target-selective inhibitor library	600	25	–	Osteosarcoma	[177]
	Green fluorescent protein	targeted anti-cancer therapy drug library	99	30	Imatinib	Triple-negative breast cancer	[178]
	Cardiac function	medicinal herbs-derived natural compounds library	347	23	Cyanidin chloride	Cardiomyopathy	[179]
	Col-12	Self-built library	614	3	Danshensu, lawsone, and sanguinarine	Collagen-related diseases	[180]
	Intestinal neutrophil accumulation and ROS level	Peach Blossom Decoction, Pulsatilla Decoction, and Gegen Qinlian Decoction	74	6	Palmatine, daidzin, oroxyloside et al	Inflammatory bowel diseases	[181]
	Sirt3	Tongmaiyangxin pills	21	5	licoisoflavone A	Cardiac hypertrophy	[182]
	Macrophage	Xuanfeibaidu Formula	104	6	Polydatin, isoliquiritin, acteoside et al	COVID-19	[183]
	α-SMA	A standard TCM compound library	344	16	Ligustrazine, glycyrrhizic acid, astragaloside iv et al	Chronic kidney disease	[184]
	YFP-CL1	Self-established TCM library	89	3	Salvianolic acid A, salvianolic acid B, and ellagic acid	Alzheimer's disease	[185]

### Computer-aided drug design

The occurrence of diseases is often related to multiple targets. Natural products can simultaneously regulate variety disease processes to achieve optimal therapeutic effects. Computer aided drug design (CADD) is a commonly used method for developing multi-target drugs, mainly divided into structure-based drug design (SBDD) (molecular docking, molecular dynamics, and virtual screening) and ligand-based drug design (LBDD) (pharmacophore model and quantitative structure–activity relationship model) [47].

#### Structure-based drug screening

SBDD is an important tool for discovering potent small molecule drugs, mainly based on the 3D structure of biomolecules or macromolecular ligand complexes for drug design, including molecular docking, pharmacophore models, and molecular dynamics simulations [48]. Virtual screening is a method of SBDD, which can be divided into receptor-based virtual screening (RBVS) and ligand-based virtual screening (LBVS) based on the presence or absence of receptor structures [49]. Among them, RBVS, also known as structure based virtual screening (SBVS), rapidly and effectively searches and discovers bioactive molecules in a large virtual compound library based on protein crystal structure or homology modeling [50, 51]. Taking the development of COVID-19 therapeutic drugs as an example, the active ingredients are docked with viral surface proteins (spike glycoprotein, ACE2, etc.) and host targets (Mpro, RdRp, etc.) to screen virus-targeted drugs (to prevent the virus from binding to host cells) and host-targeted drugs (to prevent the virus from replicating in host cells) [52, 53]. The active ingredients screened after molecular docking should be further calculated (such as molecular dynamics simulation) to increase the reliability of docking [54]. In addition, SBDD is also applied to drug development for complex diseases, involving software and case studies as shown in Table 3.

**Table 3:** Structure-based and ligand-based drug repurposing methods

Method	Main Software	Introduce	Example		
			Protein Crystal	Medicine	Refs
Molecular Docking	AutoDock(AutoDock 4, AutoDock Vina and PyRx)	Free for academic use. AutoDock applies a semi-flexible docking method with Protein flexibility to evaluate the docking results based on the binding free energy. Docking algorithm takes genetic algorithm, simulated annealing, etc. (https://autodock.scripps.edu/)	M^pro^ (PDB:6LU7), PL^pro^ (PDB:4OVZ), RdRp(PDB:6NUS),S-protein (PDB: 6VSB)	Luteolin	[186–188]
	DOCK	Free for academic use. Dock applies a semi flexible docking method to reassemble the rigid fragments of small molecules based on the geometric properties of the receptor surface for conformational search, has Protein flexibility. Dock uses a force field to score the docking results. The docking algorithm uses shape fitting, lowest energy binding. (https://dock.compbio.ucsf.edu/)	M^pro^ (PDB:6LU7)	Andrographolide	[189, 190]
	SwissDock	Free for academic use. SwissDock is a web server based on the protein ligand docking program EADock DSS. SwissDock uses a force field to score the docking results (Full fitness score and binding affinity values). Docking algorithm uses stochastic, local search and combination of broad and local search of the conformational space. (http://www.swissdock.ch.)	Nsp15(6VWW)	Sarsasapogenin, ursonic acid, ajmalicine, silymarin, novobiocin and aranotin	[191–193]
			M^pro^(6Y84)	Curcumin, demethoxycurcumin, EGCG, EGC, hesperidin et al	
	Fred	Free for academic use. FRED operates in traditional docking mode and mixed mode, which uses the structure of binding ligand and protein structure to screen molecules. It’s Docking algorithm is Exhaustive search algorithm. (https://www.eyesopen.com/oedocking)	SARS-CoV-2 S-ACE2 complex (7DF4)	HCC1,4,11	[194, 195]
	SLIDE	Free for academic use. SLIDE can accurately calculate the flexible docking of receptors, and search and calculate all the alternative conformations of receptor ligand complexes. According to the theoretical model of uniform field, the docking score is made. (https://www.schrodinger.com/)	–	–	[196]
	eHiTS	Free for academic use. eHiTS breaks the ligand into rigid segments, connects flexible chains, and independently and systematically docks each rigid segment to every possible position in the cavity. Score the docking results according to empirical and knowledge. (https://bip.weizmann.ac.il/toolbox/structure/ehits.htm)	–	–	[197]
	CDOCKER	Commercial. Based on molecular dynamics, the algorithm of annealing (semi-flexible docking) is simulated by using CHARMm force field. Score the docking results according to Force-field. (https://www.3ds.com/products/biovia/discovery-studio)	M^pro^ (PDB:6LU7)	Viomycin, 5,3′,4′-trihydroxyflavone, scutellarein, and shikonin	[198–200]
	MOE	Commercial. MOE is a comprehensive application environment and technology development platform, which supports the design of small molecular drugs and biopharmaceuticals through Conformational analysis. Has Protein flexibility. Score the docking results according to the Empirical and Force-field. (https://www.chemcomp.com/en/Products.htm)	S-protein (PDB: 6VSB)	Ginsenoside Ra2, ginsenoside Rb1, glycyrrhizic acid, berberine chloride et al	[201–203]
			M^pro^ (PDB:6LU7) S-protein (PDB:6ZCZ)	Salvianolic acid B	
	FlexX	Commercial. FlexX uses fragment growth method to consider the flexible and fast automatic docking procedure of ligand conformation, and takes free energy as the basis to evaluate docking results.Docking algorithm uses incremental and construction. (https://flexx.readthedocs.io/en/stable/)	M^pro^ (PDB:6LU7)	Mitoxantrone, leucovorin, birinapant and dynasore	[204, 205]
	GOLD	Commercial. GOLD uses genetic algorithm for docking, and the ligand is completely flexible and the protein is partially flexible when docking. With Protein flexibility. GOLD scores the docking results according to Force-field. (https://www.ccdc.cam.ac.uk/discover/blog/gold-the-all-in-one-molecular-docking-package/)	M^pro^; 3CL^pro^; PL^pro^	Flavonoids extracted; phenylethanoid glycosides	[206–208]
	Surflex	Commercial. Surflex is a fully automatic and flexible molecular docking algorithm, which combines the scoring function of Hammerhead docking system with search engine (molecular similarity based on surface) and scores the docking results with Empirical. With protein flexibility. The docking algorithm uses incremental and xonstruction. (http://www.biopharmics.com/products.html)	3CL^pro^ (PDB: 6LU7)	–	[209, 210]
	GLIDE	Commercial. GLIDE is a complete systematic search for the conformation, orientation and location space of docking ligands. Based ligand docking with energetics, Combine the conformational analysis (ChemScore function) results of the docking. (https://www.schrodinger.com/platform/products/glide/)	M^pro^	–	[211, 212]
Molecular Dynamics	GROMACS	GROMACS is suitable for biological macromolecules, supporting various common classical force fields and several less visible force fields. In addition, the software has rich built-in analysis tools and adopts a multi-level parallel approach to allocate computing tasks. (https://www.gromacs.org/)	M^pro^	Peonidin 3-O-glucoside, kaempferol 3-O-β-rutinoside, 4-(3,4-Dihydroxyphenyl)−7-methoxy-5-[(6-O-β-D-xylopyranosyl-β-D-glucopyranosyl)oxy]−2H-1-benzopyran-2-one, quercetin-3-D-xyloside, and quercetin 3-O-α-L-arabinopyranoside	[213, 214]
	AMBER	AMBER is a tool set composed of multiple components, which supports different molecular types such as protein, DNA, RNA, carbohydrate and fat, as well as a general force field GAFF and multiple water molecular models for small organic molecules. (https://ambermd.org/tutorials/)	S-protein	Withanolide D and withaferin A	[215, 216]
	CHARMM	CHARMM is a universal and flexible molecular simulation software that provides strong support for water molecule models, membrane models and implicit solvent models (such as polarization force fields). (https://academiccharmm.org/)	M^pro^	Hinokiflavone, myricetin and artemisinin	[217, 218]
	Desmond	Desmond can efficiently simulate biological systems, using algorithms and numerical techniques to simulate display solvent/membrane systems. (https://www.schrodinger.com/platform/products/desmond/)	M^pro^	Laurolitsine	[219, 220]
	LAMMPS	LAMMPS is a large-scale atomic/molecular massively parallel simulator, which can be used to simulate systems of atoms, polymer molecules, biomolecules, metals and particles. And supports a variety of coarsening models. (http://lammps.sandia.gov/)	–	–	[221]
	ESPResSo	Espresso specifically for coarse graining model. The simulation system includes polymers, liquid crystals, colloids, polyelectrolytes, ferrofluids and biological systems. (http://espressomd.org)	–	–	–
	NAMD	nAMD is a kind of dynamic simulation software for biological macromolecules (proteins, lipids, carbohydrates, nucleic acids), which is extensible. And supports an implicit solvent model based on Generalized Born model. (http://www.ks.uiuc.edu/Research/namd/)	M^pro^	Curcumin derived polyphenols	[222, 223]
	DL_POLY	At present, DL_POLY has two versions which are DL_POLY _ Classic and DL_POLY_4. It can simulate macromolecules, polymers, ionic systems and solutions by molecular dynamics. (https://www.scd.stfc.ac.uk/Pages/DL_POLY.aspx)	–	–	[224]
	CP2K	CP2K is a molecular dynamics simulation software based on the first principle, which is widely used to simulate solids, liquids, molecules, periods, materials, crystals and biological systems. (http://www.cp2k.org/)	–	–	[225]
	CPMD	CPMD is a powerful ab initio scientific simulation software, which is widely used in the research of material physics, chemistry and macromolecular biology. (http://www.cpmd.org/)	–	–	[226]

#### Ligand-based drug screening

LBDD is another widely used method in CADD, which can be used when protein crystals (such as membrane proteins) are unknown (Fig. 1f). The screening of LBDD is mainly achieved by evaluating the molecular similarity between the submitted molecules and the disease-related biologically active molecular library [55]. The pharmacophore model clarifies the space and pharmacodynamic characteristics required for the interaction between drug molecules (ligands) and specific biological target receptors, which has been widely used in the research and development of natural products. Similarly, taking COVID-19 drug development as an example, collect the structure and efficacy characteristics of small molecule anti COVID-19 drugs with precise therapeutic effects as the training set. The structure and efficacy characteristics of small molecule anti-COVID-19 drugs with definite efficacy were collected as the training set. Then the multi-dimensional pharmacophore algorithm (HipHop, LigandScout, etc.) was used to establish the pharmacophore [55, 56]. The component has multi-target activity if a compound can match multiple pharmacophores [57].

#### Others

With the rapid development of artificial intelligence (AI) technology, new research tools have been provided for the field of drug repurposing, which not only shortens the R&D cycle but also provides new options for some rare or intractable diseases [58]. Large-scale studies have confirmed that sildenafil protects brain cells and may become a new treatment for alzheimer's disease [59, 60]. Currently, AI drug repurposing includes data mining and analysis, machine learning, and computational simulation. The TxGNN model proposed by Marinka Zitnik's team uses graph neural networks and metric learning modules to achieve drug repurposing in clinical centers [32]. Secondly, the quantitative structure–activity relationship based on AI technology applies AI technologies such as deep learning and machine learning to the study of the relationship between drug molecular structure and biological activity, accelerating the development of combination drugs [61, 62]. In addition to the above methods, fragment-based virtual screening (FBVS) technology can discover molecules with higher binding efficiency through fewer fragment compounds, and has become a hot topic in drug research [63].

With the advent of the era of big data, the integration of computer simulation technology and in vitro research can quickly and accurately screen multi-target drugs and combined drugs from the natural product library [64, 65]. Whether it is a single-target drug or a multi-target drug, the process of drug research and development includes two stages: the discovery and optimization of the lead. The biggest challenge after obtaining a lead compound is to optimize the pharmacodynamics (PD) and pharmacokinetics (PK) of the drug while considering the rationality of target combination, activity balance, and target selectivity. If the activity difference of natural products to each target is too large, it is difficult to set the dosage, leading to adverse reactions or difficult to achieve the desired effect. Therefore, identifying the interaction between drugs and targets is an important research field of drug discovery.

## The mode of action of natural products

Clarification of mode of action (MOA) helps to promote drug development based on ensuring drug safety. For example, the main molecular target of the antimalarial drug quinine has yet to be identified, resulting in an unknown MOA of recognized biologically active molecules that cannot reduce its toxic response [66]. In addition, MOA provides a basis for drug combination methods, such as the significant improvement of tumor tissue destruction in all cancers through the use of multiple monoclonal antibody combinations [67]; the combination of antiepileptic drugs with different MOAs has greater effectiveness and can reduce the risk of hospitalization and emergency treatment [68]. However, the main MOA of natural products based on phenotypic screening is unknown. Drugs based on target screening rarely bind to direct targets alone, and indirect targets need to be explored to help improve the efficacy/selectivity of compounds [69]. Here we summarize the identification methods for direct/indirect drug targets.

### Direct target discovery strategy

**Fig. 2:**
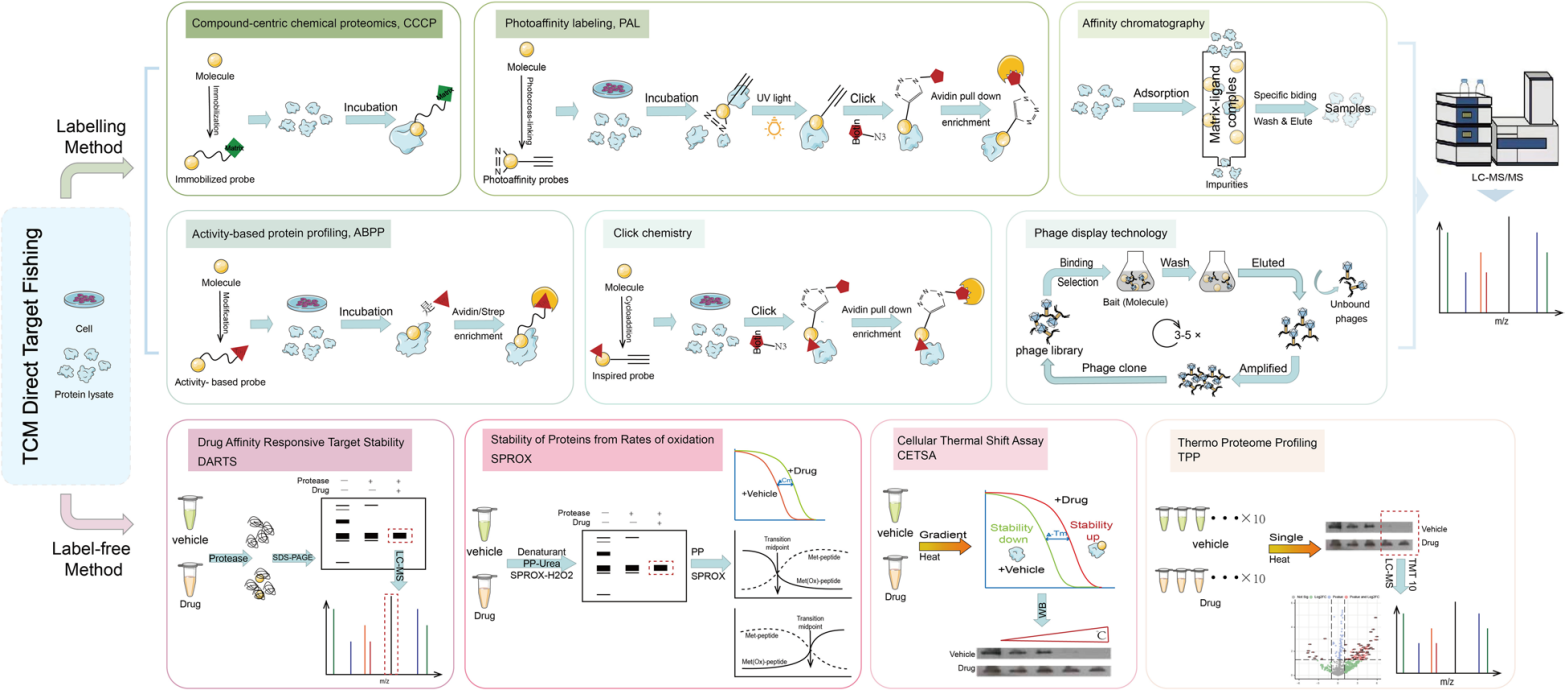
Direct targets fishing. TCM: Traditional Chinese Medicine; LC–MS/MS: Liquid Chromatography-Tandem Mass Spectrometry; UV: Ultraviolet

The direct target is the key to understanding the active substance MOA in natural products. The interaction between the “internal exposure dose” of small molecules of natural products entering the target organ and the direct target is the basis of their direct pharmacodynamic effects [70]. For example, the oncoprotein PML-RAR is the direct drug target of As2O3 in the treatment of acute promyelocytic leukemia [71]. The "target fishing" strategy is a highly operational direct target identification technique (Fig. 2), which mainly uses the principle of changing the stability of drugs after binding with target proteins to identify the possible targets of natural products, specifically divided into labeling, non-labeling and other methods (Table 4) [72]. These technologies can quickly capture target proteins that may have specific interactions with drug-active substances.

**Table 4:** Identification methods of drug direct/indirect target

	Technology	Principle	Application Characteristics	Limitation	Application	References
Labeling Method	Immobilized probe	Conventional bioactive small molecules are affixed to biocompatible inert resins, which are then used to isolate target proteins from the entire proteome	The synthesis is simple, and all adsorbed protein can be analyzed indiscriminately	The length of the connecting chain and the structural modification of small molecules can affect the binding force between the affinity probe and the target protein	Trapoxin and histone deacetylase (HDAC) specific binding	[227–229]
	Activity-Based Protein Profiling (ABPP)	Probes are synthesized by retaining the pharmacological activity of their parent molecule and subsequently incubated with live cells, lysates or tissue homogenates, and protein targets are identified using proteomic methods after binding to the target protein	Directly applied to living cells, it reflects the real drug-target interaction under cell physiological conditions	Prone to false positive results	Parthenolide modifies focal adhesion kinase 1 (FAK1)	[227, 229,230]
	Photoaffinity probe (PAL)	Chemical probes are prepared by doping photoreactive groups to covalently cross-link to adjacent amino acid residues under UV irradiation. Click chemistry-based enrichment captures labeled proteins for proteomic analysis	It has small volume and high efficiency	Binding of some non-specific proteins, affecting the accuracy of the results	Tropomyosin as a novel target of betulinic acid	[227, 231, 232]
	Click chemistry	Based on the synthesis of carbon-heteroatom bonds (C-X-C), small molecule probes are obtained by introducing orthogonal reactive groups (alkynes, alkenes, etc.) into the structure of natural products. Subsequently, the probes are bound to proteins in situ using click chemistry in cells. Finally, azide-modified reporter groups (biotin or fluorescent) are attached to the probe-target protein complexes using click reactions. And then the targets are detected, enriched and identified (SDS-PAGE)	Can be carried out in living cells; It has rapid and specific chemical coupling under aqueous conditions; It has little or no effect on the structure and pharmacological activity of small molecules	Some natural product active ingredients do not bind well to their targets; azide is toxic	Artemisinin could successfully pull-down plasmepsinl (PM I), plasmepsin II (PM II), merozoite surface protein 1 (MSP1) and actin	[227, 233–235]
	Small-molecule affinity chromatography	Using the affinity between small molecules and proteins, the small molecules are made into solid-phase adsorbent and subsequently adsorbed on the protein with affinity in the protein mixture, and finally, a suitable eluent is selected to elute the bound proteins	High affinity and specificity, suitable for complex organic systems with extremely low target product concentrations	It is difficult to identify proteins that have an affinity for this spatial structure	Isochlorogenic acid A and luteolin-7-O-glucuronide have a stronger affinity with vascular endothelial growth factor receptor 2 (VEGFR2)	[236–239]
	Phage Display Technology	Build a phage library and incubate with the target molecule, then remove unbound and non-specific phages, and use buffer to elute and separate the target bound phages. Finally, the eluted phages are infected and amplified in the bacteria	Simple, efficient, and low-cost	Three to five rounds of biological selection are required to separate specific and high affinity peptide binders	Bcl-2 was identified as paclitaxel binding protein	[240–242]
Label-Free	Drug affinity responsive target stability (DARTS)	After the drug is combined with the corresponding protein, it will make the structure of the target protein relatively stable, and the activity of the same enzymatic hydrolysis reaction will change, making it less susceptible to protease decomposition	Small molecules needn’t structural modification, have strong specificity, and can directly use original small molecule compounds or even inconspicuous compound drugs as research objects, making the operation convenient	Identifying targets with low protein abundance has limitations	Aconitine could well bind to cytosolic phospholipase A2 (cPLA2)	[50, 64,236,239,243]
	Cellular thermal shift assay (CETSA)	Incubate small molecules with cell and tissue homogenate, then undergo thermal denaturation, using the principle of target proteins becoming structurally stable after binding to drug molecules, and finally verify using protein immunoblotting	High selectivity	High drug concentrations, commonly used for target identification, are still to be determined for larger and more complex soluble proteins and membrane proteins to be determined	Direct binding of Herbacetin and serum/glucocorticoid regulated kinase 1 (SGK1)	[51, 236,244]
	Stability of proteins from rates of oxidation (SPROX)	The denaturation dependence of methionine side chain oxidation in proteins mediated by hydrogen peroxide (H_2_O_2_) is used to measure the folding free energy of proteins and the Kd value of protein ligand complexes, similar to the DARTS technique	Enables detection of direct and indirect effects of protein-ligand binding in complex biological mixtures	The rate of oxidation is susceptible to amino acid residues. Protein concentration is affected by chemical denaturants, resulting in a weak oxidation reaction that is not easily detectable	28 proteins were identified to interact with manassantin A	[236, 244–246]
	Thermal proteomic profiling (TPP)	A technique that combines the CETSA method with quantitative proteomics techniques. Using the principle that high temperature can denature proteins, cells are heated before and after administration. Subsequently, the denaturation curve was obtained through quantitative analysis of soluble proteins	Identify direct targets of action indiscriminately from intact cells or tissues. And can calculate the melting temperature (Tm) of the target protein	Only detect the interaction between small molecules and soluble proteins	Nucleolar protein 14 (Nop14) is a specific target of vioprolide A	[57, 247,248]
	Limited proteolysis mass spectrometry (LiP-MS)	LiP-MS uses the principle of limited hydrolysis to identify protein targets of small molecule drugs. After binding to the target protein, the small molecule drug will stabilize the rotein structure at the overall or local level, which greatly reduces the possibility of peptides on the protein surface being exposed and cleaved by proteases	No need to design synthetic molecular probes, use drug molecules to directly conduct binding experiments in vitro to obtain direct binding targets and their binding sites	Low abundance proteins are difficult to detect; When small molecule binding causes changes in protein structure, it is difficult to accurately locate the precise region of drug binding	The deubiquitinase UCHL3 is a direct target of farrerol	[249, 250]
Other	Proteolysis-targeting chimeras (PROTAC)	Also known as “Targeted Degradomics”. Use the ubiquitin-protease system to develop drugs for target proteins and find drug targets from degraded differential proteins	Inducing the degradation of target proteins does not require strong affinity, which is beneficial for discovering targets with weaker affinity	Off-target effects; fewer PROTAC molecules targeting “undruggable” proteins	MAFF protein is a potential target of lathyrol	[251, 252]

### Indirect target discovery strategy

More and more studies have proved that there are complex and comprehensive dialogue mechanisms between various organs. In many pathological cases, the communication between organs has changed, and the regulation of multiple organ crosstalk is the internal system regulation strategy for the treatment of disease or injury [73]. Dual-directional or multidirectional communication connections such as kidney-brain axis [74], lung-gut axis [75], gut-brain axis [76] have also been proposed in drug treatment of complex diseases or chronic diseases. Several studies have proposed that natural products regulate the physiological metabolic process through intermediary substances (such as endogenous metabolites, exosomes, intestinal flora, etc.) in the treatment of complex or chronic diseases, thus playing an indirect therapeutic role in the disease [77]. In addition, the development of indirect targets helps to avoid the technical challenges of undruggable targets and reduce the toxic side effects of drugs [78]. Therefore, disease-related networks, protein–protein interaction networks, and other biological network models can be constructed through systems biology methods. By analyzing the network topology and interactions between nodes, indirect targets can be identified.

#### Systems biology

The basic information flow in the biological system is from DNA to RNA, then to protein and finally to metabolites (genome-transcriptome-proteome-metabolome). These technologies can quickly and comprehensively grasp the pathogenesis of the disease [79]. In recent years, research on patent drug targets has turned to molecular approaches for identifying new targets based on the cellular mechanisms underlying disease phenotypes [80]. In addition to characterizing and understanding the disease, "omics" technology can also determine how drugs act at the molecular level [69, 81].

Genomic drug targets are the discovery of disease-related pathogenic genes and new drug targets through genomic technology, providing strategies for clinical disease prevention, diagnosis and treatment [82, 83]. Since the late 1970s, mRNA has been experimentally used as a potential therapeutic target, and the discovery pathways include whole genome sequencing, gene chips or RNA sequencing, genomics, etc., to identify genetic variant sites or differentially expressed genes associated with diseases. As we all know, the same gene is associated with many different indications, and the development of therapeutic drugs targeting this gene may be applicable to the diseases associated with this gene [84]. For example, 5α reductase inhibitors are used not only for prostate hyperplasia but also for hair loss [85]. In addition, genomics can also evaluate the effectiveness and specificity between compound targets [86]. By disrupting protein expression when binding to mRNA and affecting disease progression [87]. Anderson DE et al. through functional screening of the bat genome found that carolacton, an inhibitor of the novel host protein MTHFD1, potently blocked replication of several RNA viruses, including SARS-CoV-2 [88].

Transcriptome is a high-throughput characterization of RNA. Previous studies have proved that drug repositioning based on transcriptomic gene expression and target identification is an effective method to find disease-candidate drugs [89]. Jia Z et al. use transcriptomics to find the key MOA of COVID-19, including endocytosis, the lysosome and neutrophil degranulation, and repositioned two antiviral drugs, saquinavir and ribavirin [90]. In recent years, the new genome editing technology CRISPR-Cas9 and RNAi technology have provided powerful tools for target discovery and identification research. For example, DrugTargetSeqR technology combines high-throughput sequencing, computational mutation discovery, and CRISPR-Cas9 to identify drug targets [91]. And successfully revealed the mechanism of action of ispinesib and YM155 and the mechanism of drug resistance.

In addition to the direct target identification strategy of chemical proteomics, proteomics also provides unique insights into disease biology beyond the genome and transcriptome. Using proteomics techniques to evaluate protein expression profiles in health and disease or drug treatment samples, identifying targets closely related to disease occurrence, development, and treatment [92]. For example, the splicing, glycolysis and nucleotide synthesis pathways are important pathways of SARS-COV-2 replication and potential therapeutic targets, and pladienolide B, 2-DG, ribavirin and nms-873 can be used as potential therapeutic options for the COVID-19 [93]. To more accurately quantify proteins, the combination of mass spectrometry with stable isotope labeling methods (^13^C, ^15^N, ^14^N, ^2^H) strategy or unlabeled methods [94, 95]. For example, Zhang et al. [96] found that Gamabufotalin may be a new inhibitor of Hsp90 at the cellular level by using a stable isotope labeling method, thus playing an anti-cancer role. In addition, protein post-translational modifications (phosphorylation, glycosylation, acetylation, etc.) can affect the function and activity of proteins, and therapeutic drugs can be developed targeting modified enzymes, modified proteins and sites [97].

Metabolomics, as a newly developed technology in recent years, most studies small molecule substances with molecular weights within 1500 Da in cells, tissues, or biological fluids, revealing changes in the metabolic characterization of the organism [98, 99]. As an indirect or auxiliary target discovery technology, metabolomics directly reflects the organism’s function by interfering with metabolic enzymes to cause the accumulation or consumption of metabolites [100]. The discovery of regulatory targets for natural products can also draw on metabolic flow analysis and functional metabolomics developed based on metabolomics technology. Studies have shown that metabolic disorders in patients with COVID-19 are associated with the severity, and intervention of arginine, tryptophan or purine metabolism can significantly improve the excessive inflammatory response [101]. Therefore, in the MOA study of natural products, metabolomics systematically quantifies the flow distribution of metabolic networks in cells or tissues, and reveals important metabolic enzymes that cause changes in metabolic flow [102]. For example, metformin exerts anti-liver cancer effects by blocking glycolytic flux through the HIF-1α/PFKFB3/PFK1 regulatory axis [103].

#### Network pharmacology

The molecular mechanism of complex diseases is caused by abnormal intracellular regulatory networks involving interactions between multiple genes and multifunctional proteins. The "one drug, one gene, one disease" model currently hinders the innovation of complex disease treatment drugs [104]. Network pharmacology is defined as “integrating systems biology, bioinformatics, network science and other disciplines, from the perspective of system level and biological network as a whole, to analyze the molecular association between drugs and treatment objects, reveal the systematic pharmacological mechanism of drugs, to guide new drug research and development and clinical diagnosis and treatment” [105]. As a multi-component complex system, natural products enter the more complex human life system and network pharmacology provides a research strategy of "multi-component-multi-gene-multi-target-complex disease" to elucidate the complex interaction mode between complex components and multiple effects of natural products [106–108].

The drug development process is a long process, mainly involving four stages: drug research and development, preclinical research, clinical trials and new drug applications, approval for marketing and post-marketing monitoring. The real target validation (disease and drug targets) in drug research and development and the balance of drug exposure/selectivity in disease-targeted tissues and healthy tissues in preclinical research are easy to be ignore, resulting in a high failure rate of clinical drug development [109]. Among them, the identification of natural product MOA is helpful in identifying candidate drugs, and further optimizing the efficacy and specificity of its inhibitory molecular targets, so as to achieve better efficacy and reduce the off-target effect. The final characterization of natural products with clear MOA is a crucial step in determining preclinical candidate drugs. Generally speaking, the final characterization includes high-dose pharmacology, PK/PD study, dose linearity and repeated administration PK of drugs, which provide the necessary data for optimizing the first-in-human dose (FIH) of drugs [110]. Methods commonly used for extrapolation from animals to humans fall into two main categories [111]: 1) the minimum expected biological effect dose (MABEL) and no observed effect level (NOAEL), which are based on the body's treatment, tolerance characteristics, and/or pharmacological properties of drugs; 2) A quantitative pharmacology model based on mechanistic principles.

## Safe medication dosing optimization

### Traditional dose optimization strategy

There are complex interactions between drugs in the process of combined drug development, and it is difficult to find the optimal dose of clinical combined drugs by using traditional design methods. Among them, predicting the maximum recommended starting order (MRSD) has become a key node in transitioning from non-clinical to clinical drug development [107]. The traditional dose evaluation method is recommended by the FDA and European Medicines Agency (EMA), which has the NOAEL and MABEL [108, 112]. NOAEL is a commonly used estimation method to determine the FIH dose of the human body in the first clinical trial. Although the factor of safety was introduced, the differences in the characteristics of PK and PD that may exist between species and the binding characteristics of drugs and receptors were ignored, resulting in a significant deviation in the effective human dose [111, 113]. In addition, this method is based on the minimum toxicity risk rather than pharmacological activity. In some cases, it cannot accurately predict the FIH dose of drugs with strong pharmacological activity and low toxicity [114].

Since TGN1412 and BIA10-2474 events, the MABEL method for estimating MRSD by fitting PK/PD data has been put forward and continuously optimized, taking into account pharmacological dose/concentration–response curve and receptor occupancy (agonist ≤ 10%, Antagonist ≤ 90%) [108]. The MABEL evaluation method dramatically reduces the risk of clinical subjects and the estimated MRSD has good tolerance. However, potential PK differences between species were overlooked and a large amount of mechanistic data was required. And for drugs with adverse reactions originating from excessive pharmacological effects, the initial dose estimation may be too high [111, 115]. There are many differences in clinical trial design related to the physical and chemical properties, biological activity, decomposition rate, absorption, distribution, metabolism and excretion (ADME) of small-molecule drugs and large-molecule drugs. This results in different methods of inter-species scaling for these two types of classification. However, as a commonly used method of interspecific scaling, the allometric scaling method is unsuitable for drugs with insufficient overall accuracy, narrow applicability, specific species binding/distribution, or liver metabolism dependence [116].

### Dose optimization strategy based on quantitative pharmacology model

**Fig. 3:**
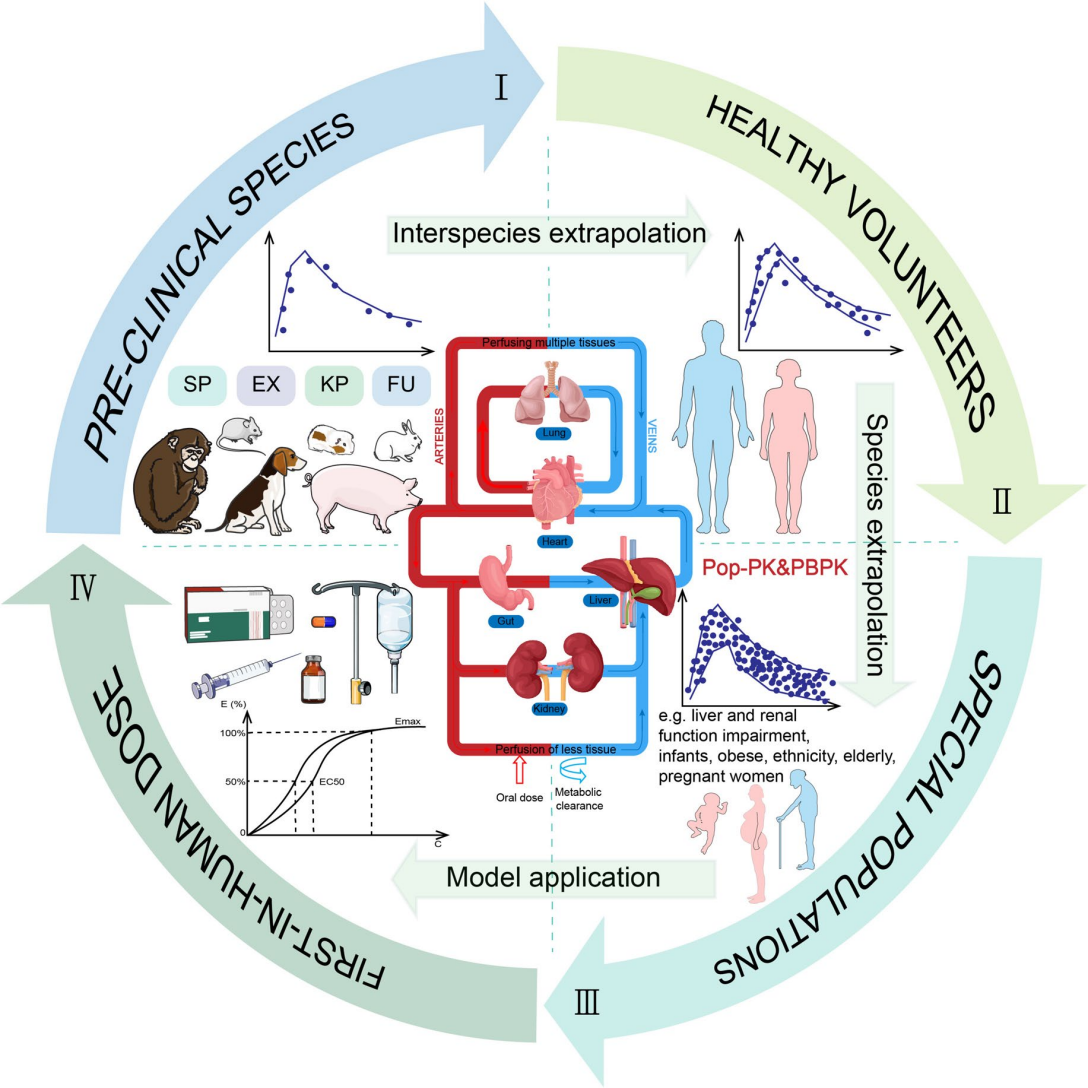
Establish physiologically based pharmacokinetic model for cross-species extrapolation of drug dose. SP: species-specific physiology like blood flow rates or organ volumes; EX: tissue-specific gene expression of enzymes and transporters; KP: kinetic parameters in active processes; FU: fraction unbound quantifying binding to plasma proteins

With the development of emerging technologies such as big biological data, computer technology, and machine learning, FDA has proposed new drug research and development and scientific evaluation based on quantitative pharmacology. This makes it possible to apply model simulation technology to study complex biological processes [117, 118]. The traditional PK/PD model is mainly used to improve the administration plan and individualized treatment of approved drugs, but this semi-mechanism PK/PD model cannot predict the drug exposure level, nor can it further predict the drug effect in different situations (between other drugs or biomarker) [119]. Therefore, FDA proposed a model physiologically based pharmacokinetic (PBPK) based on physiological, biochemical, and anatomical parameters, which simulates the ADME process of drugs in the body by connecting various tissues and organs with blood circulation as the center [120, 121] (Fig. 3). Compared with the traditional dose optimization model, PBPK not only considers the physical and chemical properties of the compound itself but also introduces physiological parameters of the body, emphasizes the different basis for describing the reaction rate constant of drug transport in and out of the room, and establishes the relationship between drug exposure and effect in target tissue [122,123]. At the same time, coupled with PD, a physiological-based pharmacokinetic/pharmacodynamic model (PBPK-PD) is constructed to characterize the systemic level of drug ADME and tissue-level drug effects, more accurately predicting the time course of drug effects. The optimal dosage regimen can be expected in various complex situations, such as combination therapy and disease complications [124]. In addition to interspecific extrapolation, PBPK provides a practical framework for in vitro in vivo extrapolation (IVIVE) [125]. For example, by integrating the in vitro data of Vero cells infected by SARS-CoV-2, the PBPK model was used to simulate the concentration of hydroxychloroquine in lung fluid under different administration schemes to explore the most effective strategy [126]. At present, PBPK has been widely used in various stages of clinical drug development, such as drug regimen adjustment for special populations [127], risk assessment [128], toxicity assessment [129], drug interaction research [130] and drug-disease interaction prediction [131].

In addition, due to race, age, gender, genetic background and many other factors, human response to drug exposure is different. Therefore, the impact of population-level variability in the dose-response relationship should be considered in the development of drug combinations [132]. As a parameter-based modeling method (bottom-up), PBPK can also be combined with population pharmacokinetics (Pop-PK) for drug dose selection [133]. Different from the classical PK, Pop-PK is a data-driven computational model that uses a "top-down" method to reflect inter-individual and intra-individual variability, identify the factors that cause pharmacokinetic variations (age, gender, race, body weight, etc.), and can be used to simulate drug concentrations in large sparse sample individuals [134, 135]. The two are combined in a complementary manner, linking exposure to clinical outcomes and addressing individual differences in the target population. A case in point is, Pop-PBPK, established by Offman E et al. [136], who confirmed that individual differences in lymphatic circulation influence the human blood volume and renal clearance rate of subcutaneous injection of polyethylene glycol peptide. In addition, the Pop-PBPK model can also provide safe and effective dosage plans for different life stages or special populations (elderly, children, pregnant women, patients with impaired kidney and liver function, etc.) [137, 138]. For example, physiological changes during pregnancy will change the drug exposure dose. Increasing the oseltamivir dose after three months of pregnancy is recommended by combining Pop-PK with the PBPK model [139]. Race difference is also one of the focuses of current drug development. The PBPK model combined with the Pop-PK model provides a solution to this problem. For instance, Feng S et al. [140] use the PBPK model to predict the PK racial sensitivity and initial dose of bitopertin. In contrast, Pop-PK was used to confirm racial differences and provide a basis for dose adjustment.

Therefore, in the study of combined administration of natural products, the PBPK model can be used to conduct multi factor correlation analysis on multiple components, tissue parameters and pharmacokinetics/efficacy at the same time [141]. Law FCP et al. [142]construct a PBPK model for tea polyphenol mixtures to predict the total concentration of tea catechin mixtures in human plasma after taking green tea or Polyphenon E (PE). In addition, the PBPK model can also be applied to the study of drug interaction in natural products. For example, according to the PBPK model, it is proved that silybin-raloxifene can be used together, and the mechanism of action is to inhibit the intestinal glucuronic acid reaction ^143^. Therefore, in the process of optimizing the dosage of natural products, the PBPK-PD model has the following advantages compared to the traditional PK/PD model: firstly, considering human ADME, dose optimization is directly carried out from the drug concentration of the target cells or organs in the human body; secondly, integration the PK results of multiple-components to reflect the comprehensive effects of various components and targets in natural products; thirdly, it compensates for the biases caused by clinical small sample trials and patient physiology; fourthly, it can overcome species differences and to some extent replace animal experiments and bioequivalence experiments, accelerating calculation speed and reducing research costs.

## Multidisciplinary research framework for the treatment of natural products of COVID-19

Since the beginning of the COVID-19 pandemic, preclinical/clinical studies of hundreds of potential drugs have been carried out, but only a few small-molecule antiviral drugs (such as remdesivir) and 11 kinds of monoclonal antibodies have been approved for clinical use to treat COVID-19. Natural products play a vital role in the prevention and treatment of this epidemic situation. Internationally recognized innovative drugs should have novel structures, new targets, mechanisms therapeutic uses and so on. This leads to the slow development process and high consumption of innovative drugs, and drug repurposing has gradually become a hot spot for common and rare diseases in treatment [144]. For instance, drugs such as chloroquine and remdesivir show good antiviral activity. Finding drugs or combinations to treat COVID-19 at different pathological stages from natural products provides a new perspective for developing COVID-19 drugs. However, there are 2 key problems in natural products and synergetic mechanism: first, the active substances lack drug action mode research, which cannot improve the success rate of drug development; Second, the current ADME research on natural products lacks multi-component integration and "in vitro in vivo" correlation model, which cannot provide data for the optimization of combination drugs, leading to the weak risk control ability of clinical trials.

But the aforementioned single technology can only solve some of the problems. HTS, HCS and CADD provide the initial chemical basis for the development of natural products. However, the screening results differ significantly from the overall structure of the biological system and cannot reflect the comprehensive pharmacological effects of the drug, and CADD is highly dependent on the quantity and quality of available data and the predictive/discriminative ability to underlying algorithms. Therefore, the combination of HTS and CADD has the advantages of rapid screening and optimization of drugs, improving the efficiency and accuracy of drug design, and speeding up the drug development process. However, the natural products identified as candidates for disease treatment are multi-target drugs that affect multiple systems in organisms (direct binding and indirect regulation). Omics technology can mine robust data and identify disease/natural product targets and mechanisms of action. However, the results of using only one method to identify natural product targets are susceptible to factors such as selectivity, specificity and biochemical or physiological correlation [100]. Therefore, according to the structural characteristics of candidate natural products, integrating multi omics methods to fully mine data significantly improves the ability to decipher complex drug target associations [145]. In addition, effective human dose prediction is a key factor in drug development, and traditional dose optimization methods cannot represent drug exposure in target organs or tissues. At the same time, the FDA issued draft guidelines to encourage the pharmaceutical industry to consider underrepresented races and populations (elderly, children, pregnant women, lactating women, and patients with comorbidities) in the early stages of drug development [146]. Model-informed drug development (MIDD) is a quantitative model based on preclinical and clinical data that helps to enhance clinical trial design/efficacy and ultimately achieve optimal dosing in individual patients, including underrepresented populations [147]. Among them, PBPK, as a component of MIDD, can simulate drug interactions and evaluate organ/tissue exposure based on the clinical situation of the initial study and outside the population, providing the best dose for individuals [148].

**Fig. 4:**
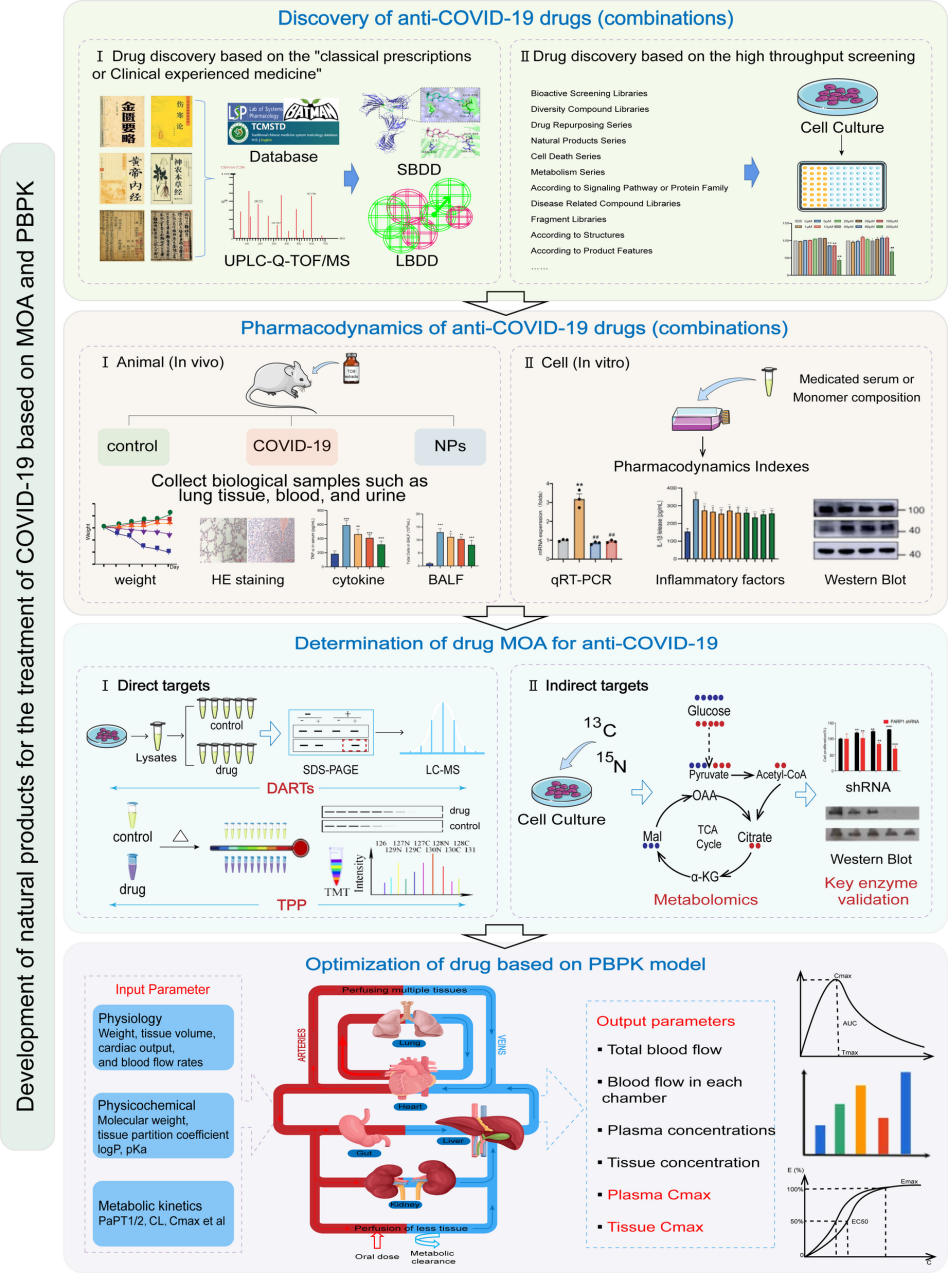
Development of natural products for the treatment of COVID-19 based on MOA and PBPK

With the development of technology, interdisciplinary research strategies and technical means have been proposed for the research and development of innovative drugs. Therefore, we offer to use new drug discovery technologies such as MOA and PBPK to conduct research on COVID-19 natural products from the perspective of “distinguishing substances, identifying patterns, and determining dosages” (Fig. 4). First, the antiviral activity of the existing natural products library extracts was tested in vitro with the help of HTS technology and CADD technology, and more professional keynote screening methods can be adopted, namely physiological screening [149]. Then, on the basis of clarifying the pharmacodynamics of active natural products, with the help of metabolomics, chemical synthesis and mass spectrometry detection technology, target discovery of active ingredients (direct and indirect targets) in complex organisms was carried out to clarify the MOA of natural products. Further classifying the “candidate natural products” with different MOAs, according to the pharmacological weight and dose-response relationship of different “candidate natural products”, provides the basis for the formulation of the optimization scheme of combined drugs. PBPK model can build a “in vitro-in vivo” bridge of candidate natural products, and the in vitro dose ratio can be inferred from the in vivo dose of target organs. Specifically, the pharmacokinetics and tissue distribution of combined natural products and different monomers in vivo can be studied through in vivo experiments to obtain the parameters required for PBPK modeling (PK, tissue distribution, excretion data, etc.). The animal PBPK model of natural products (monomers) was constructed by using the "top-down" strategy, and the PBPK model of combined natural products was further constructed by integrating and analyzing the PBPK model parameter values of different natural products. The optimal ratio of combined natural products was determined by PBPK model, and the modeling parameters were replaced with human-specific values to achieve different dose extrapolation and species extrapolation.

## Conclusion and future perspective

Drug research and development is at the core of the pharmaceutical industry, aiming to find safe and effective therapeutic drugs. Single-target drugs can accurately target a single target of a disease, thereby blocking the biological activity of the target. Compared with multi-target drugs, they have advantages such as clear mechanism of action, relatively smooth development process, and predictable safety. In recent years, studies have shown that single-target drugs have potential side effects in the treatment of complex/chronic diseases (such as tumors, depression, diabetes, etc.), such as narrow application range, easy to develop drug resistance, insufficient efficacy and poor safety. With the development of medical technology and further research on disease pathogenesis, it is found that multi-target drug therapy can make up for the limitations of single-target drug therapy for complex/chronic diseases. A large number of natural products have been proven to have obvious clinical therapeutic advantages. The compatibility optimization of candidate natural products with clear activity can greatly improve the accessibility of new drug discovery, such as the combination of arsenic tetrasulfide, indirubin and tanshinone IIA in the treatment of leukemia [150]; The combined extract of polyphenols in tea (veregen™) has become the first botanical drug approved by FDA [151]. As the starting point for the development of natural product therapies, these studies focus on multi-target drug development based on multi-component combinations, which not only promotes the discovery of innovative drugs, but also improves the feasibility of developing multi-target drugs based on natural products.

In recent years, natural product research has increasingly shown the characteristics and advantages of multidisciplinary cross-integration, mainly focusing on the discovery and isolation of natural products, structural modification and synthesis of natural products, and the mechanism of action and efficacy evaluation of natural products [152]. However, drug discovery is a systematic process, and multi-target drug development faces huge challenges and problems. Mainly reflected in the following 4 aspects: First, disease-related biological networks are complex, and which combination of targets can produce the best therapeutic effect has become a major problem; secondly, the synthesis and design success rate of multi-pharmacophore molecules is low.; thirdly, whether multi-target drugs specifically bind to the expected target also affects the optimization of pharmacokinetics and pharmacodynamics; finally, multi-target drugs are highly heterogeneous in clinical research and have complex trial designs (it is necessary to consider multiple factors such as dose optimization and efficacy), and safety is difficult to monitor. Based on the existing problems in current multi-target drug research, this article proposes a research strategy that combines advanced technologies for drug discovery such as phenotypic screening, MOA and PBPK. Provide strategies for the research of multi-target drugs derived from natural products from 2 aspects: the determination of real targets and the balance of multi-target activities, the impact of target synergy on dose, and the balance between clinical dose/efficacy/toxicity. This strategy uses HCS platforms to concentrate resources on in-depth research and optimization of potential multi-target natural products, or uses computer-assisted multi-target drug design to optimize the affinity and stability of multi-target drugs. Subsequently, on the basis of clarifying the in vivo drug efficacy, direct target and indirect target mining technologies are integrated to comprehensively explore the complex mechanisms of diseases and drugs, improve the efficiency and accuracy of target identification, and eliminate off-target effects. These strategies ignore the balance of drug exposure/selectivity between disease-targeted tissues and healthy tissues. For example, the exposure/selectivity of remdesivir, an antiviral drug for COVID-19, at a dose of 100 mg in the lung may be too low, while the concentration in the kidney may be too high. Therefore, the optimal dose is high drug exposure (low dose) in diseased tissues and the minimum drug exposure (high dose) in healthy tissues [109]. Therefore, by establishing a PBPK model to balance the efficacy and safety of multi-target drugs, the optimal dosage of multi-target drugs can be accurately adjusted based on the in vivo pharmacokinetic parameters and efficacy of natural products.

Although this article proposes a development strategy for natural product multi-target drugs, it is not sufficient to solve all the problems. First of all, the genetic, functional or compositional heterogeneity of healthy and diseased tissues has also brought significant challenges to drug discovery and development [153]. Single-cell omics or whole-genome sequencing provides a new direction for patient stratification and personalized treatment. For example, Deng G et al. [154] used a research strategy that combines single-cell multi-omics with target confirmation to find that cycloastragenol (Astragalus membranaceus) can directly target and downregulate tissue cathepsin B, enhancing anti-tumor immunity. In addition, Tang C et al. [155] proposed comboSC, which stratifies individual patient samples through single-cell RNA sequencing to identify synergistic drug/small molecule combinations or small molecules that can be paired with immune checkpoint inhibitors. In addition, although the PBPK model balances the efficacy and safety of multi-target drugs to a certain extent. However, the reference values established by the PBPK model require a large number of experimental measurements, and some species' parameters are difficult to obtain. Meanwhile, the introduction of the 3R principle (i.e., replacement, reduction, and refinement) and the species differences between animals and humans have led to greater drug sensitivity. On this basis, a research model combining the PBPK model and organ chips was proposed [156]. Organ chips can obtain some data that are impossible or difficult to obtain in traditional experiments, further improving the accuracy and efficiency of PBPK simulation results. Therefore, innovative research technologies should be combined in drug development strategies to effectively promote the transformation of new drugs from concept to clinic.

## Data Availability

This manuscript has no associated data.

## Abbreviations

FDA: US Food and Drug Administration
HTS: High throughput screening
HCS: High content screening
CADD: Computer-aided drug design
QSAR: Quantitative structure–activity relationship
AI: Artificial intelligence
VS: Virtual screening
SBDD: Structure-based drug design
LBDD: Ligand-based drug design
PD: Pharmacodynamics
PK: Pharmacokinetics
MOA: Mode of action
MABEL: Minimum expected biological effect dose
NOAEL: No observed effect level
MRSD: Maximum recommended starting order
EMA: European Medicines Agency
ADME: Absorption, distribution, metabolism and excretion
PBPK: Physiologically based pharmacokinetic
PBPK-PD: Physiological-based pharmacokinetic/pharmacodynamic model
IVIVE: In vitro in vivo extrapolation
PopPK: Population pharmacokinetics
PE: Polyphenon E
MIDD: Model informed drug development
STORM: Spatial Temporal Operative Real Metabolomics
